# Balancing plant conservation and agricultural production in the Ecuadorian Dry Inter-Andean Valleys

**DOI:** 10.7717/peerj.6207

**Published:** 2019-02-13

**Authors:** Catalina Quintana, Marco Girardello, Henrik Balslev

**Affiliations:** 1 Facultad de Ciencias Exactas, Escuela de Ciencias Biológicas, Pontificia Universidad Católica del Ecuador, Quito, Pichincha, Ecuador; 2 Center for Ecology, Evolution and Environmental Changes, Azorean Biodiversity Group, Azores University, Azores, Portugal; 3 Department of Bioscience, Aarhus University, Aarhus, Denmark

**Keywords:** Endemics, Zonation, Agriculture, Ecuador

## Abstract

**Background:**

Conserving both biodiversity and ecosystem services is a major goal of the Convention on Biological Diversity. Hotspots for biodiversity in the Andes significantly overlap with areas with dense human populations that sustain their economy through agricultural production. Therefore, developing management forms that reconcile food provisioning services—such as agriculture—with biodiversity conservation must be addressed to avoid social conflicts and to improve conservation in areas where biodiversity co-occurs with other ecosystem services. Here, we present a high-resolution conservation plan for vascular plants and agriculture in the Ecuadorian Dry Inter-Andean Valleys (DIAV) hotspot. Trade-offs in conserving important areas for both biodiversity and agriculture were explored.

**Methods:**

We used a dataset containing 5,685 presence records for 95 plant species occurring in DIAVs, of which 14 species were endemic. We developed habitat suitability maps for the 95 species using Maxent. Prioritization analyses were carried out using a conservation planning framework. We developed three conservation scenarios that selected important areas for: biodiversity only, agriculture only, and for both biodiversity and agriculture combined.

**Results:**

Our conservation planning analyses, capture 33.5% of biodiversity and 11% of agriculture under a scenario solely focused on the conservation of *biodiversity*. On the other hand, the top 17% fraction of the *agriculture only* scenario captures 10% of biodiversity and 28% of agriculture. When biodiversity and agriculture were considered in combination, their representation varied according to the importance given to agriculture. The most balanced solution that gives a nearly equal representation of both biodiversity and agriculture, was obtained when agriculture was given a slightly higher importance over biodiversity during the selection process.

**Discussion:**

This is the first evaluation of trade-offs between important areas for biodiversity and agriculture in Ecuadorian DIAV. Our results showed that areas with high agricultural productivity and high biodiversity partly overlapped. Our study suggests that a land-sharing strategy would be appropriate for conserving plant diversity and agriculture in the DIAV. Overall, our study reinforces the idea that friendly practices in agriculture can contribute to biodiversity conservation.

## Introduction

Ecosystem services provide several benefits to local communities including water purification, carbon sequestration, and crop production. These services form the basis for the livelihoods of many people who depend on them (Haines-Young & Potschin, 2009). However, ecosystem services are increasingly threatened by water pollution, soil degradation, and the loss of plants and animals (Balmford et al., 2002; Evans et al., 2003). When such conflicts appear, the need to find a balance must be sought by our society in general and policy makers in particular. Ecosystem services have attained a central importance in all major economic and political agendas (Turner & Daily, 2008). One of the key targets on the International Convention of Biological Diversity (Aichi target 11) states that at least 17% of all terrestrial areas, especially areas of particular importance for biodiversity and ecosystem services, should be preserved by 2020.

In tropical mountains economic activities such as agriculture are common practices of local people. Moreover, in densely populated areas that coincide with hotspots biodiversity suffer the pressure of agricultural expansion (Laurance, Sayer & Cassman, 2014). Agriculture has often been seen as incompatible with conservation, and as a main driver of biodiversity loss (Geist & Lambin, 2002). In the Andean mountains agriculture dates back thousands of years (Maass et al., 2005) and great expansion of cultivated areas is expected to occur in the near future. Agricultural areas in Ecuadorian, Colombian, and Peruvian Dry Inter Andean Valleys (DIAVs) overlap with high biodiversity that now survives only in small patches of vegetation.

**Figure 1:**
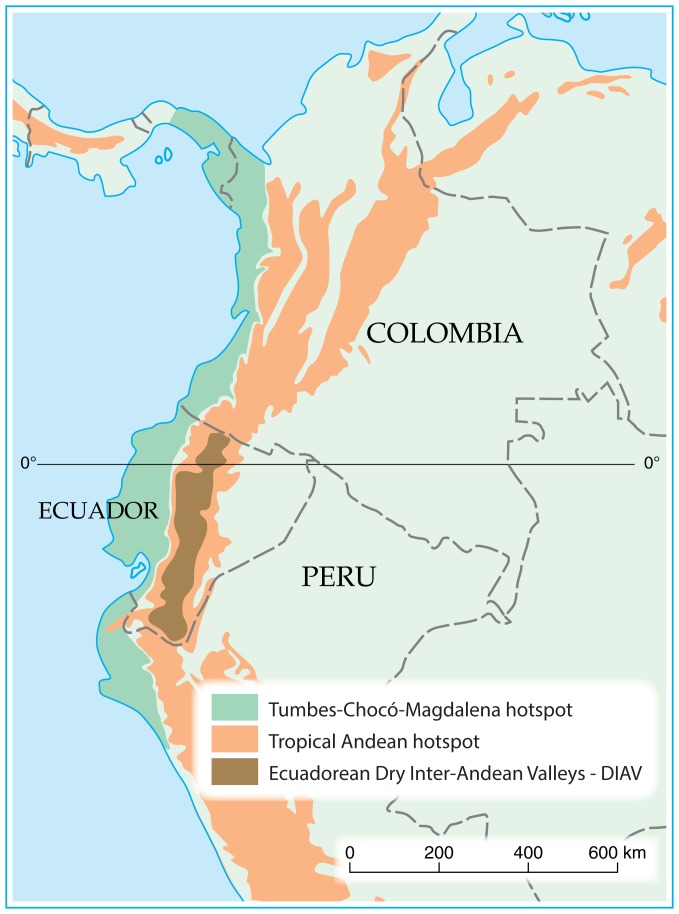
Map of northern South America with hotspot areas: Tropical Andes and Tumbes-Chocó-Darien.

Ecuadorian DIAVs (Fig. 1) cover approximately 59,000 km^2^ between the western and eastern cordilleras of the Andes, and they are unique in diversity and endemism of plants and animals (Lozano, 2002; Weigend, 2002; Torres-Carvajal, 2009). One of every three species of flowering plants in DIAVs is endemic (Quintana et al., 2016). Such a high endemism is attributed to climatic seasonality that promotes ecological differences between habitats (Loaiza, 2013), as well as topography and mountain barriers (Weigend, 2004; Quintana et al., 2016, 2017) that isolate valleys (Särkinen et al., 2012). Both northern and southern DIAVs are part of important South American biodiversity hotspots such as the “Tropical Andes” and the “Tumbes-Chocó-Magdalena” (Brummitt & Lughadha, 2003) (Fig. 1). Ecosystem services associated with dry ecosystems and therefore with DIAVs include protection of freshwater sources, agricultural and pastoral goods, maintenance of soil fertility, biodiversity, climate regulation, flood control, scenic beauty, provision of medicinal plants, provision of fuel wood, and construction materials. Green vegetation also provides shade, which associated with cooling winds is much appreciated by the inhabitants of these dry areas (Maass et al., 2005).

Agriculture is a vital provisioning service for human well-being and a key component of the global economy. Nearly 40% of the Earth’s terrestrial surface is covered by agroecosystems and because agricultural practices can reduce biodiversity through multiple pathways, agricultural land use and biodiversity conservation have traditionally been viewed as incompatible. Much threatened biodiversity lives in agricultural areas, but the trade-offs between biodiversity and farming exists and depend on shared landscape methods (Mehrabi, Ellis & Ramankutty, 2018). These areas, if extensively managed for food production could support high levels of biodiversity (Durán, Duffy & Gaston, 2014). Thus, failure to consider agricultural areas would leave many species unprotected including some falling within protected areas. This reality does not apply only to the western world but is important also in developing countries. It is important to identify adequate land-management techniques to promote conservation in countries like Ecuador (Wright, Lake & Dolman, 2012).

In Ecuador, agriculture is a key component in the local economy, and as much as 40% of the local population in the DIAVs depend on agriculture which in turn generates 20% of the gross national product for valleys in the north of the country, 15% for central valleys and 21% for southern valleys (Ministerio de Coordinación de la Producción Empleo y Competitividad, 2011). However, since 1990 a boom of green houses for floriculture has abruptly changed the inter-Andean landscape. Some 4,000 ha were established by 1999 in northern DIAVs which strongly affected the economy and cultural perspective of the region (Knapp, 2010). The Ecuadorian national state budget increased by 3% thanks to the flower industry (Ministerio de Coordinación de la Producción Empleo y Competitividad, 2011). The demand for water increased as a consequence of the agricultural expansion because soils derived from volcanic ashes need irrigation to sustain agriculture (Wilson et al., 2010).

As a consequence of this pressure from agriculture, the native vegetation is now reduced to small remnants in inaccessible areas and regrows along the edges of agricultural lands. These patches now remain as witnesses of a past continuous forest (Quintana et al., 2016). DIAVs are amongst the most degraded ecosystem on earth (Gentry et al., 1995), with less than 10% of their original area remaining in Ecuador (Ministerio Del Ambiente & MAGAP, 2015). Today in addition to agriculture, urban expansion, and wood extraction are the common drivers of present day vegetation cover (Quintana et al., 2016). Several large inter-Andean cities like Quito (1,6 million inhabitants), Cuenca (333,000 inhabitants), and Loja (180,000 inhabitants) continue to expand (INEC, 2014). DIAVs in Ecuador have provided charcoal for energy in local communities for more than a century, particularly using hard wood trees like *Acacia macracantha* (Quintana, 2010).

Under this scenario it is a challenge to find appropriate conservation strategies that can preserve important areas for both biodiversity and agriculture. The specific aims of our study were (i) to identify critical areas for plant conservation using a spatial prioritization technique and (ii) to explore synergy trade-offs between plant conservation and agriculture in these areas.

## Methods

### Study area

Ecuador’s DIAVs are covered by deciduous and semi-deciduous vegetation. During the rainy season the forest is green and leafy, while in the dry season it shows the trees’ bare and spiny stems. Shrubs are the most speciose life-form followed by herbs and trees (Quintana et al., 2016). The deforestation that these DIAVs have suffered is extremely high and the average annual reduction is 1.4% of the area (Manchego et al., 2017).

### Biodiversity data

The DIAV flora include both exclusive and nonexclusive species. For analytical purposes, we defined those species as “exclusive” that have a ranges of 30,000–60,000 km^2^ and those with ranges >60,000 km^2^ we call “non-exclusive” and they mostly occur in DIAVs and in addition in neighboring ecosystems such as mountain forest, páramos, Amazonian and Pacific lowlands. We used a dataset containing 5,685 presence records for 95 plant species occurring in DIAVs. The dataset is a compilation of records from the QCA Herbarium database (Quito-Ecuador), the AAU database (Aarhus-Denmark), and the Tropicos database (Missouri-USA). We built a dataset by extracting occurrence records for the taxa related to dry ecosystems in Ecuador. All the records of the data set were georeferenced a priori. This dataset contains 56% of exclusive species and 44% of nonexclusive species. Fifteen percent of the species were endemic to Ecuador and of these 12 species were exclusive to DIAVs <30,000 km^2^ (concentrated just in northern or southern valleys), whereas two endemic species had wide range distributions (>60,000 km^2^) in Ecuador.

The 95 species were chosen to give a good representation of DIAVs species range patterns going from “exclusive” species which are small range to “nonexclusive” which are widely distributed species (Quintana et al., 2017). We also included DIAV species that are threatened by local communities; 64% of the nonexclusive species are used, as a source for charcoal production, firewood, construction materials, or as forage which is a destructive use that can drive the species to extinction (De La Torre et al., 2008; Quintana, 2010). Furthermore, these species are likely to be the ones that suffer most from climate change, because their original ecosystems, which are montane or Amazonian lowlands, will become drier (Phillips et al., 2009).

### Agricultural data

We use a one km^2^ global layer created by IIASA-IFPRI (http://cropland.geo-wiki.org) that provides the percentage of cropland for each grid cell. This dataset was derived by combining multiple satellite data sources such as GlobCover 2005 and MODIS as well as national maps from mapping agencies and other organizations. The final map was validated using crowd-sourced accuracy checks, to provide an improved record of total cropland extent as well as field size for the study area (Fritz et al., 2015).

### Habitat suitability modeling

We developed habitat suitability maps for the 95 species using Maxent, which is a widely-used approach to species distribution modeling based on Bayesian maximum entropy estimation (Phillips, Anderson & Schapire, 2006). We kept the default settings when running our models, with the exception of the number of replicates that was set to 10. In order to obtain a reliable evaluation of the model, we randomly split the occurrence data into two subsets, using 70% of records to calibrate the model and the remaining 30% to evaluate the model. This procedure was repeated across the 10 replicates and final consensus predictions were obtained by averaging the predictions across the 10 runs. For each replicate, we evaluated the predictive performance of the models by calculating the area under curve (AUC). Although the AUC is extensively used as metric for evaluating model performance, it has drawbacks (Lobo, Jiménez-Valverde & Real, 2008). We also computed the true skill statistic (TSS) as complementary method to the AUC. The TSS has the advantage that it does not depend on species prevalence (Allouche, Tsoar & Kadmon, 2006). As we did not have independent data to validate our models, we carried out a manual validation based on expert opinion and our own knowledge of the area, and of what is known on the distribution of selected species. The validation consisted in examining whether the models had under-predicted or over-predicted the distribution of the species for areas where these species are known to occur.

### Prioritization analysis

We used the results from the habitat suitability modeling exercise to identify important areas for the 95 species. Prioritization analyses were carried out using the Zonation framework (Moilanen et al., 2005, 2014). Zonation produces a hierarchical prioritization of the landscape based on the value of a set of sites. The algorithm proceeds by removing the least valuable cells in a landscape while minimizing the loss rate of biodiversity and connectivity. The order of cell removal gives a landscape zoning with the most important areas remaining last. The output of the zonation analysis, hereafter termed a solution, is the ranking of each site, allowing for the identification of the most important areas for species persistence. Zonation can be initialized using different removal rules depending on the conservation planning goals. We used the additive-benefit function-cell-removal rule, where conservation value is additive across species, favoring sites with high species richness while considering species’ proportional distribution in a given cell. We regarded this removal rule as more appropriate because our aim was to establish compromises among biodiversity and agriculture, implying that overall efficiency is desirable and that a degree of substitution between biodiversity and agriculture must be allowed. A key feature of Zonation is that it can take species conservation value into account by using a species weighting procedure, which stresses the selection of high-value cells toward species of conservation concern. We assigned weights to plant distributional layers and agriculture on the basis of three conservation scenarios. We first developed two separate scenarios for biodiversity and agriculture. Weights were assigned to species according to the classification developed by León-Yánez et al. (2011). In brief, this classification ranks endemic species according IUCN criteria. If a species is not endemic we assigned it a score of 1. The scores thus were: non-endemic = 1, non-evaluated = 2, data deficient = 3, least concern = 4, near threat = 5, vulnerable = 6, and endangered = 7. In the agriculture only scenario, we assigned agriculture a weight of 1 and the biodiversity features weight of 0, which means that they are ignored by the analyses. In the third scenario, we developed a multi-criterion analysis in which we prioritized areas aimed at reducing conflict between priority areas for biodiversity and agriculture. In this scenario plant layers were weighted in such a way that their total weight was equal to 1. This was achieved by dividing the species’ individual weights by the total weight across all the species; that is, 1/168 for the non-endemics, 2/168 for non-evaluated, 3/168 for data deficient, 4/168 for species of least concern, 6/168 for vulnerable species, 7/168 for endangered species. Agriculture was also assigned a weight of 1. Finally, we explored the consequences for biodiversity representation when agriculture is considered a trade-off or a conservation feature. This was achieved by carrying out nine additional multi-criteria analyses (Moilanen et al., 2011), where the weight for biodiversity was kept constant at 1 and the weight for agriculture was varied from −8 to +8.

## Results

### Habitat suitability models

The models performed well when tested against the validation data. AUC values can be interpreted as indicating reasonable to good model discrimination ability according to the subjective guidelines of Swets (1988) and Heikkinen et al. (2006). All the models had a sufficiently accurate predictive performance (AUC >0.70) (Table S1) to be used in the zonation analyses.

#### Spatial prioritization

**Figure 2:**
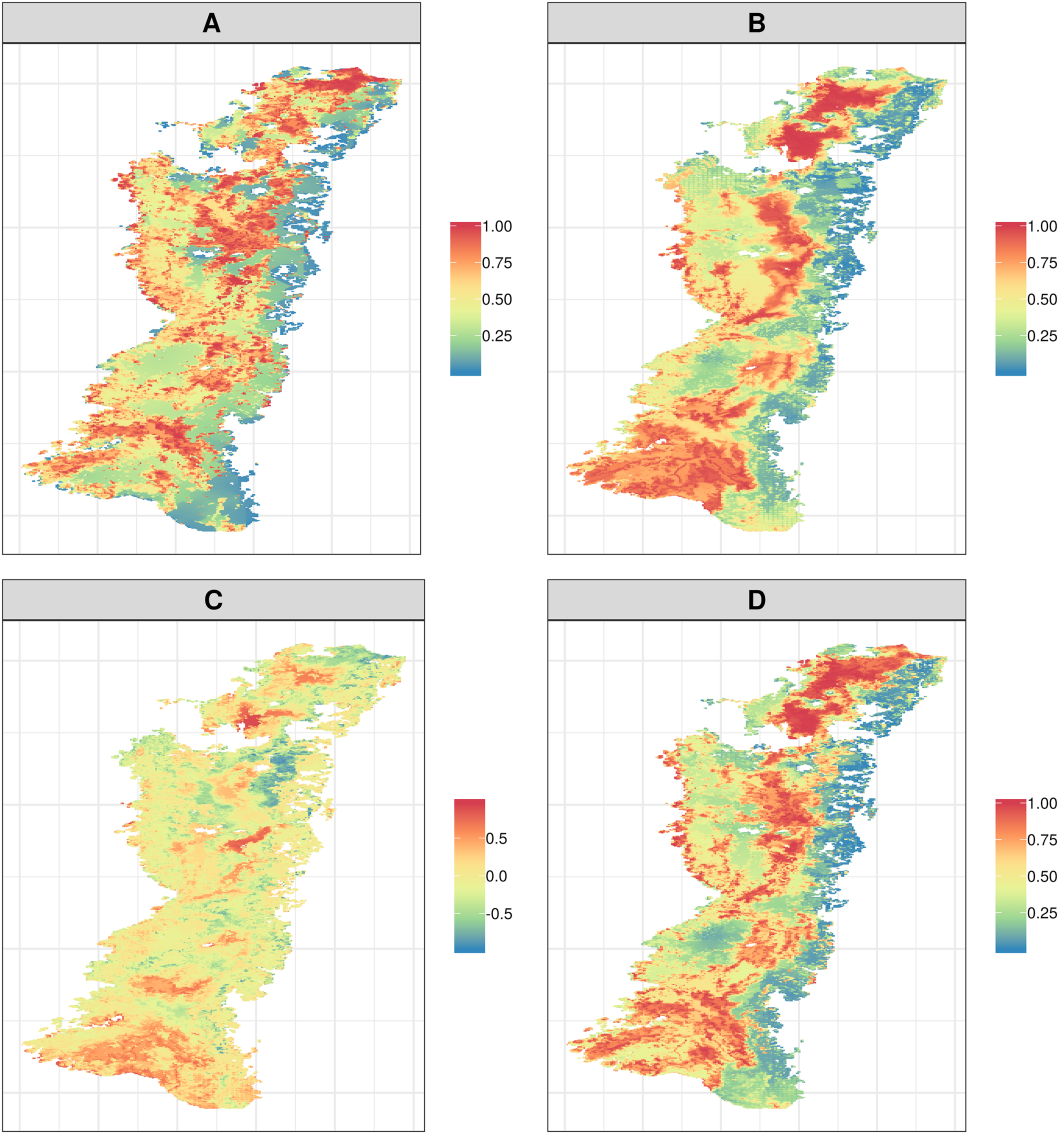
Priority ranking maps showing areas that would be most suitable for the conservation of biodiversity and agriculture. Separate maps showing priorities for biodiversity (A), agriculture (B), the difference between the biodiversity and agriculture solutions (C), and biodiversity and agriculture combined together (D).

The top 17% fraction of the biodiversity only scenario captured 33.5% of biodiversity and 11% of agriculture. High priority biodiversity areas (areas that represented a high species occurrence) were located at the western parts of the northern valleys, the eastern parts of central valleys, and at the western parts of southern valleys (Fig. 2A). In the agriculture scenario, the top 17% of land captures 10% of biodiversity and 28% of agriculture. High priority agriculture areas occurred in the extreme north of northern valleys, to the east of the central valleys, while in the south they were sparse (Fig. 2B).

When subtracting biodiversity from agriculture, the top 17% highest priority areas differed considerably (Fig. 2C). High priority areas for this strategy almost disappear in the Ecuadorian DIAVs, and the remaining areas are found in northern remnants, east central valleys and southern valleys. A high evidence of low priorities areas appeared all through DIAVs extension, showing the presence of densely populated areas, main cities (Quito, Cuenca, Loja) and agricultural lands (Fig. S1).

When both biodiversity and agriculture were considered together in the multi-criteria solution the top 17% highest priority areas differed from those ranked only with the biodiversity content. Therefore, areas identified with highest priorities for biodiversity were not always most beneficial in terms of agriculture especially for southern DIAVs. High priorities areas for this strategy are found especially in northern, central valleys to the east and to east and west in southern valleys (Fig. 2D).

**Figure 3:**
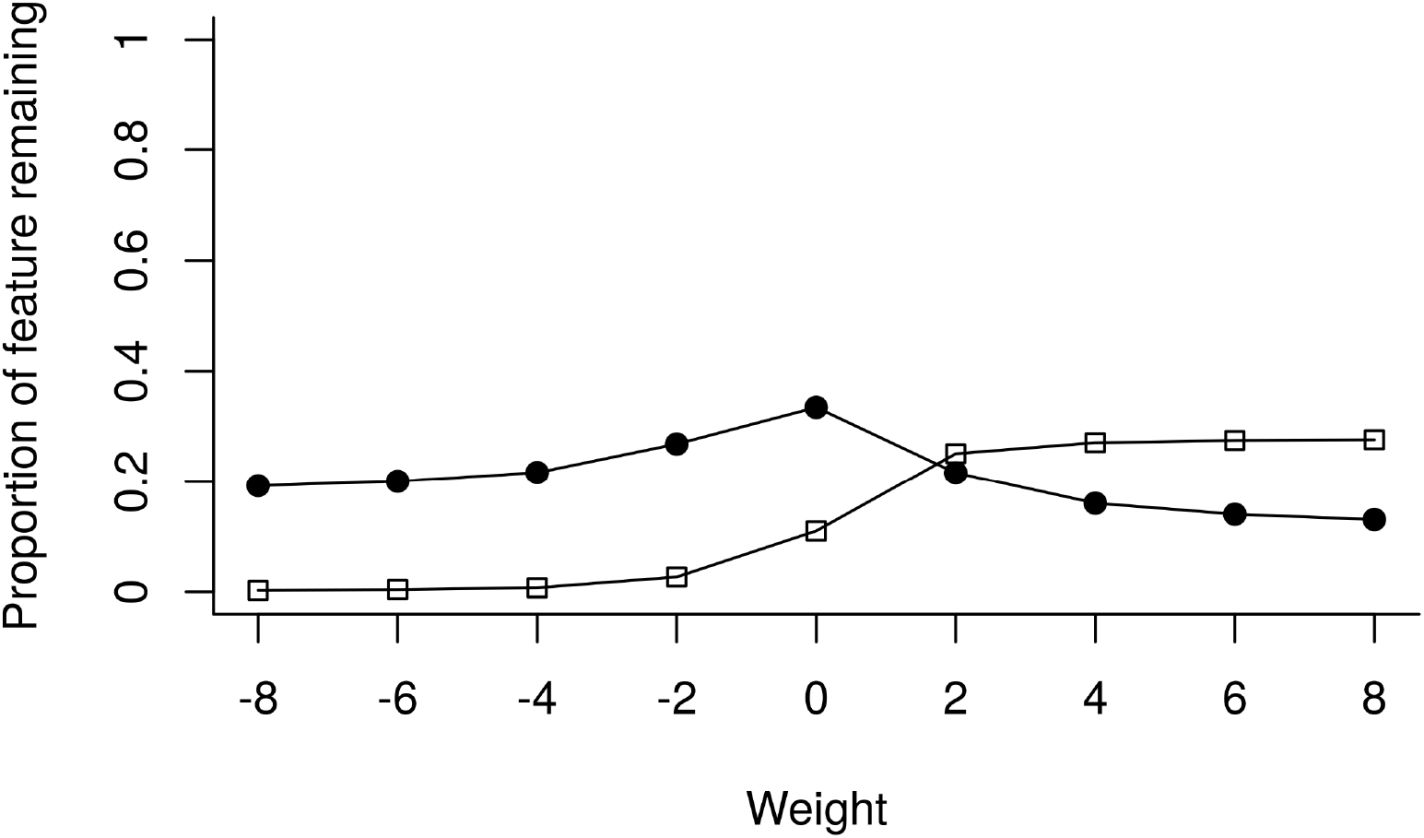
Sensitivity analysis when varying the weights of agriculture. The *x* axis depicts the relative weightings given to agriculture (biodiversity was kept constant at 1.0). The *y* axis represents the proportion of the feature remaining in the top 17% of the landscape.

The results of the sensitivity analysis shows that when agriculture was assigned a negative weight, biodiversity always had a higher representation in the top 17% fraction of the landscape. As an example when agriculture had a weight of −8, biodiversity had a value of 19% and agriculture a value of 0.002%. This trend was maintained even when agriculture was assigned a weight of 0 (and hence ignored) giving values of 33% for biodiversity and 11% for agriculture. When agriculture was assigned a positive weight the levels of representation of this feature were generally higher than biodiversity, with exception of the solution where agriculture had a weight of 2 when the representation levels of biodiversity and agriculture were nearly equal (21% and 25%, respectively) (Fig. 3).

## Discussion

Conservation prioritization analysis is essentially a multi task challenge (Watts et al., 2009). The starting point is to not only to include biodiversity but also other ecosystem services that can have positive or negative effects (Adams, Pressey & Naidoo, 2010). In this study, we evaluated, for the first time, important areas for biodiversity and agriculture in Ecuadorian Dry-Inter Andean Valleys. Our results showed that areas of high agricultural productivity and high biodiversity partly overlapped.

The top 17% fraction of the biodiversity only scenario showed higher priorities in southern valleys in Ecuador (Fig. 2A). This coincides with previous studies that showed that southern DIAVs are the largest ones and better preserved in terms of biodiversity (Aguirre & Kvist, 2005; Quintana et al., 2016). When the 17% of the only agriculture scenario was presented in the map it highlighted areas of intensive agriculture and richer soils in DIAVs (central–northern valleys) (Gray, 2009) (Fig. 2B). This scenario showed the importance of agriculture as an ecosystem service in Ecuador as it generates 20% of the gross national product (Ministerio de Coordinación de la Producción Empleo y Competitividad, 2011). However, agriculture has reduced native vegetation remnants by 50% compared to the original cover (Mosandl et al., 2008). Furthermore, it is an ecosystem service with negative side effects such as being water demanding and polluting (Evans et al., 2003; Geiger et al., 2010). Under the pressure of intensive agriculture native vegetation remains only in inaccessible and drier areas or surrounding agricultural fields.

When agriculture and biodiversity were considered together, northern valleys had more priority areas, suggesting that biodiversity and agriculture co-occur in these areas. In the areas surrounding agricultural fields with crops of maize, tomatoes, bean or fruit trees like avocados or citrus plants, native vegetation can easily grow (Quintana, 2010; Quintana et al., 2016). Biodiversity loses priority areas in southern valleys, most likely because of the presence of less fertile soils and fewer cultivated areas.

Regarding the difference map (biodiversity-agriculture) large populated cities like Quito, Ecuador’s capital, as well as Cuenca, Loja, Ibarra and others are located within the boundaries of the DIAVs. As stated before northern and central Ecuadorean DIAVs have fertile soils where irrigation is used to enhance agricultural production. In central DIAVs 2% of the land have indurated soils due to deforestation of native vegetation for agriculture and pasture (Custode et al., 1992). The tiny areas of agreement occur in private lands, areas of difficult access and areas were soils are clayey and not suitable for agriculture like in southern Ecuador (Fig. 2C).

It should, however, be noted that the spatial priorities revealed by our analyses were based on 95 species out of a total flora of 317 species in the study area (Quintana et al., 2016). While this is a limited sample we believe that as a first assessment it may show the general pattern. This can be extended to cover the rest of the species and we propose that future studies should also include more than one ecosystem service such as water, soils, and pollinators. Pollination services are particularly important for the area, while global layers for pollination and other ecosystem services already exist, these are generally available at a coarse resolution. Specific high-resolution data products should be developed in order to enable the production of improved conservation plans. The ever-increasing availability of remote sensing data offers a promising avenue for mapping multiple ecosystem services at unprecedented spatial and temporal resolutions (De Araujo Barbosa, Atkinson & Dearing, 2015).

## Conclusions

Our study demonstrates that in Ecuador conservation efforts aimed at maintaining both agriculture and plant biodiversity should be concentrated in northern and southern DIAVs. There, several areas that are important for biodiversity coincide with areas important for agriculture, which shows that land sharing will be the best strategy to optimize conservation areas for both biodiversity and agriculture. As DIAVs vegetation is mostly preserved in small patches, it will be hard to promote the conservation of these areas through a land sparing strategy. Instead friendly practices in agriculture should be introduced to preserve ruderal vegetation surrounding agricultural fields. These patches of native vegetation have the potential to secure several ecosystem services in this hotspot area. By preserving biodiversity and ecosystem services the goal of primary importance in the global conservation agenda will be fulfilled as well as the Aichi Biodiversity Target 11.

Studies that aim to optimize the selection of biodiversity and other features such as ecosystem services, exemplified by the present study, are urgently needed especially in biodiversity hotspots. It is imperative to continue the recollection of more data on both threatened and not threatened species for comprehensive analysis and also to benefit from the whole gamut of ecosystem services that the region is providing.

## Supplemental Information

10.7717/peerj.6207/supp-1 Supplemental Information 1 List of species used in the analysis with the AUC value, AUC SD=standard deviation, True skill statistic values mean (TSS) and TSS SD = standard deviation.

10.7717/peerj.6207/supp-2 Supplemental Information 2 Population density map of Ecuadorian Dry Inter Andean Valleys.

10.7717/peerj.6207/supp-3 Supplemental Information 3 List of species used in the analysis.

## Additional Information and Declarations

### Data Availability

The following information was supplied regarding data availability: Raw data are available in the Supplementary Table and at Zenodo: Catalina Quintana, Marco Girardello, & Henrik Balslev. (2018). Balancing plant conservation and agricultural production in the Ecuadorian Dry Inter-Andean Valleys. DOI 10.5281/zenodo.1524035.
