# Supplementary material for: Balancing plant conservation and agricultural production in the Ecuadorian Dry Inter-Andean Valleys
Source: PeerJ. 2019 Feb 13;7:e6207. doi: 10.7717/peerj.6207 (PMC6377594; doi:10.7717/peerj.6207)
Supplement: Supplemental Information 1 [file peerj-07-6207-s001.docx]

**Table 1**

| Species | AUC | AUC SD | TSS.MEAN | TSS.SD |
| --- | --- | --- | --- | --- |
| *Abutilon ibarrense* Kunth | 0.9792 | 0.0098 | 0.90667 | 0.06439232 |
| *Acacia macracantha* Humb. & Bonpl. ex Willd. | 0.9415 | 0.015 | 0.80815 | 0.03990236 |
| *Acalypha padifolia* Kunth | 0.7626 | 0.0821 | 0.72224 | 0.13095618 |
| *Aegopogon cenchroides* Humb. & Bonpl. ex Willd. | 0.7806 | 0.0597 | 0.65001 | 0.11431475 |
| *Agave americana* L. | 0.8503 | 0.0401 | 0.79093 | 0.1137764 |
| *Alnus acuminata* Kunth | 0.808 | 0.0732 | 0.61 | 0.1197219 |
| *Aloysia scorodonioides* (Kunth) Cham. | 0.9453 | 0.0343 | 0.85 | 0.12692955 |
| *Alternanthera porrigens* (Jacq.) Kuntze | 0.883 | 0.0264 | 0.66317 | 0.04076387 |
| *Ambrosia arborescens* Mill. | 0.8899 | 0.0298 | 0.79999 | 0.09779924 |
| *Arcytophyllum thymifolium* (Ruiz & Pav.) Standl. | 0.8521 | 0.0381 | 0.6862 | 0.08200149 |
| *Aristida adscensionis* L. | 0.9282 | 0.0181 | 0.77907 | 0.06780102 |
| *Astrolepis sinuata* (Lag. ex Sw.) D.M. Benham & Windham | 0.8886 | 0.0696 | 0.85 | 0.11486707 |
| *Baccharis latifolia* (Ruiz & Pav.) Pers. | 0.8349 | 0.0366 | 0.70624 | 0.10644868 |
| *Buddleja bullata* Kunth | 0.8702 | 0.0756 | 0.86365 | 0.11537896 |
| *Buddleja lojensis* E.M. Norman | 0.8535 | -1 | 0.78 | 0.22010099 |
| *Calamagrostis viridiflavescens* (Poir.) Steud. | 0.8774 | 0.0389 | 0.88 | 0.07888106 |
| *Capsicum rhomboideum* (Dunal) Kuntze | 0.9315 | 0.0238 | 0.7857 | 0.05581595 |
| *Cestrum peruvianum* Willd. ex Roem. & Schult. | 0.8363 | 0.0661 | 0.79231 | 0.07299826 |
| *Cheilanthes bonariensis* (Willd.) Proctor | 0.9136 | 0.0349 | 0.74544 | 0.0574867 |
| *Cheilanthes myriophylla* Desv. | 0.9294 | 0.0203 | 0.832 | 0.0454117 |
| *Cleistocactus icosagonus* (Kunth) F.A.C. Weber | 0.8694 | 0.0596 | 0.7875 | 0.10290908 |
| *Cleistocactus sepium* (Kunth) F.A.C. Weber | 0.9209 | 0.024 | 0.78529 | 0.07724568 |
| *Clethra fimbriata* Kunth | 0.8724 | 0.062 | 0.82501 | 0.11419147 |
| *Commelina diffusa* Burm. f. | 0.8283 | 0.0384 | 0.68236 | 0.0794003 |
| *Coriaria ruscifolia* L. | 0.8601 | 0.0557 | 0.73 | 0.13374935 |
| *Coursetia dubia* (Kunth) DC. | 0.8793 | 0.0392 | 0.85 | 0.09461097 |
| *Coursetia gracilis* Lavin | 0.922 | 0.0402 | 0.8375 | 0.08436857 |
| *Croton elegans* Kunth | 0.834 | 0.0632 | 0.86668 | 0.12613099 |
| *Croton menthodorus* Benth. | 0.9116 | 0.0441 | 0.83532 | 0.09113417 |
| *Croton wagneri* Müll. Arg. | 0.8685 | 0.0504 | 0.73332 | 0.1073259 |
| *Cyathostegia mathewsii* (Benth.) Schery | 0.9093 | 0.0349 | 0.81666 | 0.09816366 |
| *Dalea coerulea* (L. f.) Schinz & Thell. | 0.8976 | 0.0328 | 0.69375 | 0.11580785 |
| *Dalea humifusa* Benth. | 0.9053 | 0.0438 | 0.77856 | 0.07100223 |
| *Digitaria insularis* (L.) Fedde | 0.9186 | 0.0245 | 0.80667 | 0.05840219 |
| *Dodonaea viscosa* Jacq. | 0.9201 | 0.0246 | 0.77367 | 0.07148123 |
| *Duranta triacantha* Juss. | 0.7868 | 0.0752 | 0.76 | 0.13498971 |
| *Echeveria quitensis* (Kunth) Lindl. | 0.9054 | 0.0418 | 0.79444 | 0.06440314 |
| *Echinopsis pachanoi* (Britton & Rose) Friedrich & G.D. Rowley | 0.7645 | 0.0916 | 0.82 | 0.12292726 |
| *Eragrostis cilianensis* (All.) Vignolo ex Janch. | 0.8522 | 0.0558 | 0.73125 | 0.09793883 |
| *Eragrostis ciliaris* (L.) R. Br. | 0.8097 | 0.0958 | 0.725 | 0.1844662 |
| *Eragrostis lurida* J. Presl | 0.9407 | 0.0273 | 0.83025 | 0.04109018 |
| *Eragrostis mexicana* (Hornem.) Link | 0.8989 | 0.0299 | 0.77057 | 0.08962334 |
| *Evolvulus argyreus* Choisy | 0.9234 | 0.032 | 0.92 | 0.07888106 |
| *Furcraea andina* Trel. | 0.8992 | 0.0343 | 0.79999 | 0.12171004 |
| *Iresine diffusa* Humb. & Bonpl. ex Willd. | 0.812 | 0.0734 | 0.74287 | 0.09033749 |
| *Juglans neotropica* Diels | 0.8625 | 0.0577 | 0.7375 | 0.10944938 |
| *Kalanchoe daigremontiana* Raym.-Hamet & H. Perrier | 0.909 | 0.0295 | 0.95556 | 0.05737179 |
| *Kalanchoe fedtschenkoi* Raym.-Hamet & H. Perrier | 0.8329 | 0.0568 | 0.79414 | 0.11516934 |
| *Lantana camara* L. | 0.9129 | 0.0313 | 0.80002 | 0.07026581 |
| *Lantana rugulosa* Kunth | 0.8682 | 0.0324 | 0.67499 | 0.10120661 |
| *Lycianthes lycioides* (L.) Hassl. | 0.9361 | 0.0228 | 0.77098 | 0.05147469 |
| *Mimosa albida* Humb. & Bonpl. ex Willd. | 0.875 | 0.0337 | 0.69566 | 0.08198314 |
| *Mimosa quitensis* Benth. | 0.9055 | 0.0312 | 0.78333 | 0.05522656 |
| *Muehlenbeckia tamnifolia* (Kunth) Meisn. | 0.8072 | 0.0531 | 0.65384 | 0.11607826 |
| *Nassella ibarrensis* (Kunth) Lægaard | 0.8186 | 0.1029 | 0.8125 | 0.13501543 |
| *Nassella mucronata* (Kunth) R.W. Pohl | 0.8905 | 0.042 | 0.75218 | 0.11235031 |
| *Onoseris hyssopifolia* Kunth | 0.9091 | 0.0543 | 0.82666 | 0.10035936 |
| *Onoseris speciosa* Kunth | 0.8355 | 0.0484 | 0.75713 | 0.09640406 |
| *Opuntia cylindrica* Engelm. | 0.9454 | 0.0234 | 0.78824 | 0.10074036 |
| *Opuntia ficus-indica* (L.) Mill. | 0.8376 | 0.0455 | 0.73157 | 0.10062847 |
| *Opuntia pubescens* J.C. Wendl. ex Pfeiff. | 0.9637 | 0.0231 | 0.92692 | 0.04604932 |
| *Opuntia quitensis* F.A.C. Weber | 0.857 | 0.0571 | 0.80714 | 0.10672788 |
| *Opuntia soederstromiana* Britton & Rose | 0.918 | 0.0289 | 0.80769 | 0.04758045 |
| *Oreopanax ecuadorensis* Seem. | 0.8822 | 0.0441 | 0.93079 | 0.06733326 |
| *Oreopanax rosei* Harms | 0.829 | 0.06 | 0.78 | 0.11352924 |
| *Otholobium mexicanum* (L. f.) J.W. Grimes | 0.8173 | 0.058 | 0.66 | 0.10633049 |
| *Oxalis peduncularis* Kunth | 0.8534 | 0.0532 | 0.73639 | 0.09039359 |
| *Paspalum azuayense* Sohns | 0.8301 | 0.0467 | 0.80525 | 0.07039287 |
| *Passiflora manicata* (Juss.) Pers. | 0.852 | 0.0591 | 0.7375 | 0.08740074 |
| *Pavonia sepium* A. St.-Hil. | 0.8825 | 0.0513 | 0.79231 | 0.08921169 |
| *Pellaea ovata* (Desv.) Weath. | 0.7562 | 0.0905 | 0.75714 | 0.17879998 |
| *Peperomia galioides* Kunth | 0.8169 | 0.0531 | 0.59 | 0.12649111 |
| *Phaedranassa schizantha* Baker | 0.8325 | 0.06 | 0.75 | 0.26352314 |
| *Pilea microphylla* (L.) Liebm. | 0.7937 | 0.1059 | 0.80002 | 0.11967541 |
| *Piper barbatum* Kunth | 0.7494 | 0.0771 | 0.5625 | 0.11023964 |
| *Pleopeltis thyssanolepis* (A. Braun ex Klotzsch) E.G. Andrews & Windham | 0.8753 | 0.046 | 0.75457 | 0.09629491 |
| *Puya aequatorialis* André | 0.9056 | 0.0338 | 0.94 | 0.13498971 |
| *Sapindus saponaria* L. | 0.886 | 0.0389 | 0.66667 | 0.13606916 |
| *Schinus molle* L. | 0.9147 | 0.0287 | 0.81766 | 0.09784656 |
| *Setaria cernua* Kunth | 0.9483 | 0.024 | 0.83 | 0.07527727 |
| *Sida cordifolia* L. | 0.8934 | 0.0579 | 0.93334 | 0.11942891 |
| *Sida poeppigiana* (K. Schum.) Fryxell | 0.8544 | 0.0451 | 0.75 | 0.15811388 |
| *Siparuna muricata* (Ruiz & Pav.) A. DC. | 0.8976 | 0.0436 | 0.72 | 0.12292726 |
| *Solanum asperolanatum* Ruiz & Pav. | 0.7749 | 0.0689 | 0.70715 | 0.09189428 |
| *Sporobolus indicus* (L.) R. Br. | 0.8502 | 0.0461 | 0.6825 | 0.06977145 |
| *Streptosolen jamesonii* (Benth.) Miers | 0.8667 | 0.0472 | 0.75332 | 0.08915848 |
| *Talinum paniculatum* (Jacq.) Gaertn. | 0.9609 | 0.0201 | 0.9357 | 0.07105139 |
| *Tara spinosa* (Feuillée ex Molina) Britton & Rose | 0.9061 | 0.0328 | 0.8 | 0.12018504 |
| *Tecoma stans* (L.) Juss. ex Kunth | 0.9211 | 0.0255 | 0.77917 | 0.0922336 |
| *Tessaria integrifolia* Ruiz & Pav. | 0.8498 | 0.066 | 0.85557 | 0.17409606 |
| *Tillandsia incarnata* Kunth | 0.9468 | 0.0222 | 0.89232 | 0.09029458 |
| *Tillandsia recurvata* (L.) L. | 0.9148 | 0.0469 | 0.88335 | 0.08051915 |
| *Tillandsia secunda* Tillandsia secunda | 0.8687 | 0.0814 | 0.81251 | 0.07150931 |
| *Vallea stipularis* L. f. | 0.7647 | 0.0757 | 0.53331 | 0.1340529 |
| *Verbena litoralis* Kunth | 0.8908 | 0.0255 | 0.82141 | 0.08415985 |
