# Supplementary figures and images for: Balancing plant conservation and agricultural production in the Ecuadorian Dry Inter-Andean Valleys
Source: PeerJ. 2019 Feb 13;7:e6207. doi: 10.7717/peerj.6207 (PMC6377594; doi:10.7717/peerj.6207)

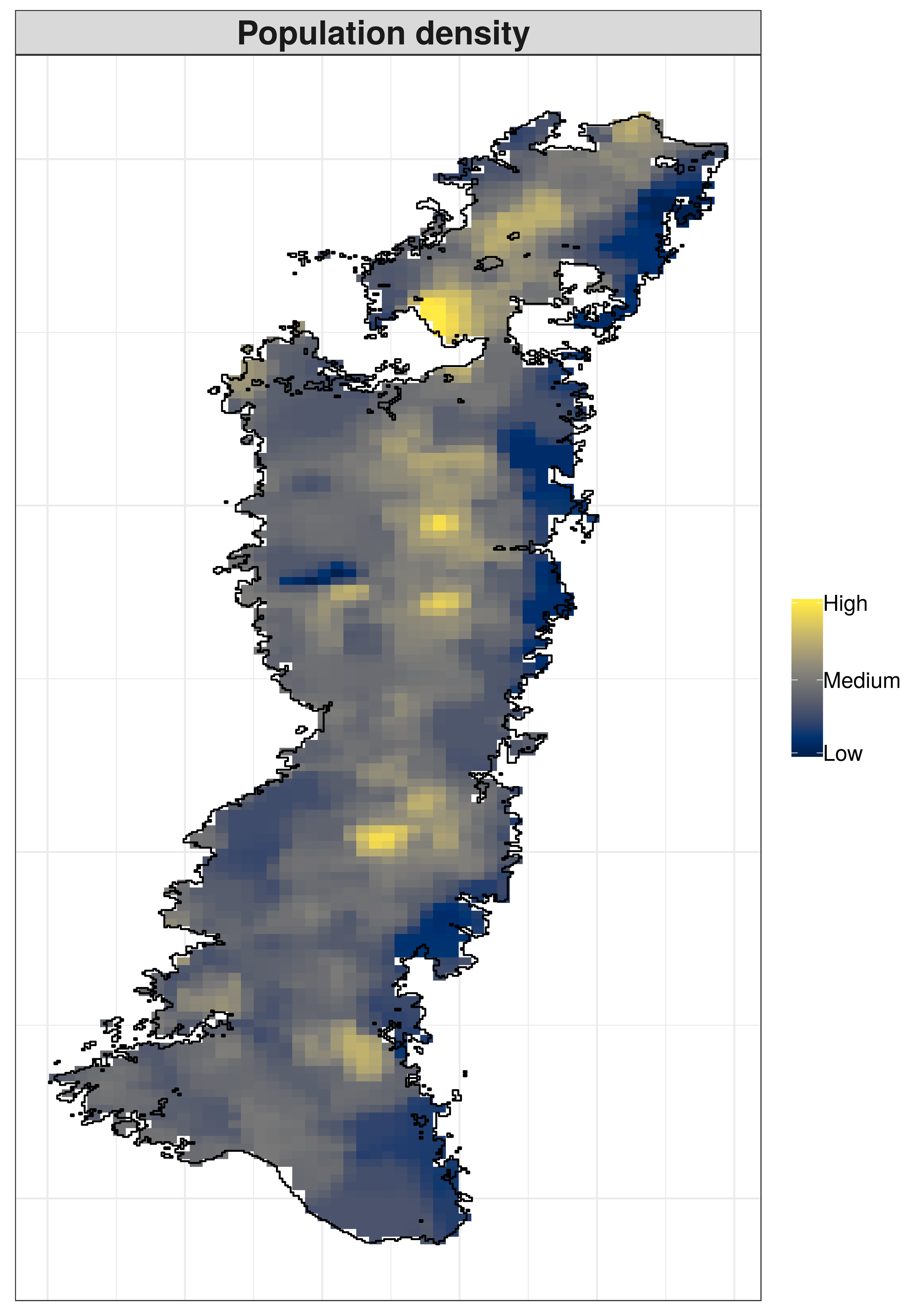

Supplement: Supplemental Information 2 [file peerj-07-6207-s002.png]
